# Clinical exome sequencing is a powerful tool in the diagnostic flow of monogenic kidney diseases: an Italian experience

**DOI:** 10.1007/s40620-020-00898-8

**Published:** 2020-11-23

**Authors:** Tiziana Vaisitti, Monica Sorbini, Martina Callegari, Silvia Kalantari, Valeria Bracciamà, Francesca Arruga, Silvia Bruna Vanzino, Sabina Rendine, Gabriele Togliatto, Daniela Giachino, Alessandra Pelle, Enrico Cocchi, Chiara Benvenuta, Simone Baldovino, Cristiana Rollino, Roberta Fenoglio, Savino Sciascia, Michela Tamagnone, Corrado Vitale, Giovanni Calabrese, Luigi Biancone, Stefania Bussolino, Silvana Savoldi, Maurizio Borzumati, Vincenzo Cantaluppi, Fabio Chiappero, Silvana Ungari, Licia Peruzzi, Dario Roccatello, Antonio Amoroso, Silvia Deaglio

**Affiliations:** 1 Department of Medical Sciences, University of Turin, via Santena 19, 10126 Turin, Italy; 2 Immunogenetics and Transplant Biology Service, Città della Salute e della Scienza University Hospital, Turin, Italy; 3 Service of Genetic Counseling, San Luigi Gonzaga University Hospital, Orbassano, Turin, Italy; 4 Department of Clinical and Biological Sciences, University of Turin, Turin, Italy; 5 Pediatric Nephrology Dialysis and Transplantation Unit, Città della Salute e della Scienza University Hospital, Turin, Italy; 6 Nephrology and Dialysis Unit (ERKnet Member)-CMID, Center of Research of Immunopathology and Rare Diseases, San Giovanni Bosco Hospital, Turin, Italy; 7 Nephrology and Dialysis Unit ASL CN1, Cuneo, Italy; 8 Nephrology and Dialysis Unit, Ordine Mauriziano di Torino, Turin, Italy; 9 Nephrology and Dialysis Unit ASL AL, Alessandria, Italy; 10 Renal Transplantation Unit ‘A. Vercellone,’ Division of Nephrology Dialysis and Transplantation, Città della Salute e della Scienza University Hospital, Turin, Italy; 11 Nephrology and Dialysis Unit ASL TO4, Turin, Italy; 12 Nephrology and Dialysis Unit of Verbania ASL VCO, Verbano Cusio Ossola, Verbania, Italy; 13 Nephrology and Kidney Transplantation Unit, Maggiore Della Carità University Hospital, Novara, Italy; 14 Nephrology and Dialysis Unit ASL TO3, Turin, Italy; 15 Struttura Semplice Genetics and Molecular Biology, ASL CN1, Cuneo, Italy

**Keywords:** Next-generation sequencing, Chronic kidney failure, Transplantation, Renal monogenic disease

## Abstract

**Background:**

A considerable minority of patients on waiting lists for kidney transplantation either have no diagnosis (and fall into the subset of *undiagnosed* cases) because kidney biopsy was not performed or histological findings were non-specific, or do not fall into any well-defined clinical category. Some of these patients might be affected by a previously unrecognised monogenic disease.

**Methods:**

Through a multidisciplinary cooperative effort, we built an analytical pipeline to identify patients with chronic kidney disease (CKD) with a clinical suspicion of a monogenic condition or without a well-defined diagnosis. Following the stringent phenotypical and clinical characterization required by the flowchart, candidates meeting these criteria were further investigated by clinical exome sequencing followed by in silico analysis of 225 kidney-disease-related genes.

**Results:**

By using an ad hoc web-based platform, we enrolled 160 patients from 13 different Nephrology and Genetics Units located across the Piedmont region over 15 months. A preliminary “remote” evaluation based on well-defined inclusion criteria allowed us to define eligibility for NGS analysis. Among the 138 recruited patients, 52 (37.7%) were children and 86 (62.3%) were adults. Up to 48% of them had a positive family history for kidney disease. Overall, applying this workflow led to the identification of genetic variants potentially explaining the phenotype in 78 (56.5%) cases.

**Conclusions:**

These results underline the importance of clinical exome sequencing as a versatile and highly useful, non-invasive tool for genetic diagnosis of kidney diseases. Identifying patients who can benefit from targeted therapies, and improving the management of organ transplantation are further expected applications.

**Electronic supplementary material:**

The online version of this article (10.1007/s40620-020-00898-8) contains supplementary material, which is available to authorized users.

## Introduction

The importance of genetic contributions in the development chronic kidney disease (CKD) is underlined by several observations: (1) inherited CKD (IKD) represents a high percentage of all CKDs [1–3], (2) the presence of a first-degree relative with end stage kidney disease (ESKD) confers a sevenfold increased risk of developing kidney failure [4], and (3) approximately 20–30% of patients report a positive family history of CKD in either a first- or second-degree relative [5, 6]. Thus, IKD represents one of the leading causes of CKD in children and adults, resulting in an increased risk of mortality, the need for organ transplantation, and high health care costs.

In the paediatric and young adult subset of patients, monogenic diseases represent up to 20% of patients who develop CKD before 25 years of age, with a variable diagnostic yield considering the different CKD categories [7–9].

Changes in DNA sequence are usually single nucleotide variants (SNVs) and small insertions or deletions (indels), but larger deletions or insertions called copy number variants (CNVs) may also occur, particularly in syndromic children.

There are several monogenic inherited diseases that cause CKD, including developmental disorders, cystic and non-cystic ciliopathies, and glomerular and tubulo-interstitial diseases [10].

Establishing a genetic diagnosis strongly impacts patient management and prognosis [8, 11, 12], both by influencing treatment choices, as is the case for focal segmental glomerulosclerosis (FSGS), and by providing access to specific drugs, as is the case of vasopressin 2 antagonists for patients with autosomal dominant polycystic kidney disease (ADPKD). For these reasons, genetic testing is increasingly utilized in clinical nephrology due to accessibility of next generation sequencing (NGS) technologies [13, 14], which are non-invasive and cost-effective, and are becoming part of the diagnostic flow for several diseases, due to their decreasing costs, high throughput abilities and reduced sequencing times [15]. In this context, NGS technology enables us to simultaneously investigate hundreds of genes, thus opening up the possibility to rapidly identify genetic factors that underlie IKDs.

This study reports on the set up of an easy-to-use and accessible genetic testing platform which can be used to characterize undiagnosed cases of CKD eligible for NGS testing. Specifically, through this analytical pipeline, we aimed at (1) confirming diagnoses, particularly for patients in whom a monogenic condition was suspected, (2) finding the genetic cause of previously undiagnosed diseases, (3) identifying patients who could benefit from targeted therapies and (4) improving the management of organ transplantation, particularly in the living donor setting.

## Materials and methods

### Patients

This project relies on the multidisciplinary collaboration between the ImmunoGenetics and Transplant Biology Service (IGTS) of the Città della Salute e della Scienza University Hospital, the Centre of Research of Immunopathology and Rare Diseases-CMID, Centre of Coordination of the Interregional Network of Rare Diseases of Piedmont and Aosta Valley (San Giovanni Bosco Hub Hospital), the local ERK-net Member (Nephrology and Dialysis Unit, San Giovanni Bosco Hub Hospital and University of Turin), the Paediatric Nephrology Dialysis and Transplantation Unit (Regina Margherita Children’s Hospital) and the Medical Genetics Unit at the San Luigi Gonzaga University Hospital.

The study included 160 patients, recruited in 13 nephrology or genetic counselling services across the Piedmont Region (north-west Italy, with a population of approximately 4,356,000), and coordinated by the IGTS between September, 2018 and December, 2019. The IGTS performed genetic testing, while recruiting centres are reported in Table 1. Overall, these centres follow > 3100 patients on dialysis and approximately 2500 transplanted patients (detailed data are available at www.trapiantipiemonte.it).

**Table 1:** NGS cohort

Recruitment centre	N. of cases (n = 160)	Sex n (%)		Age at recruitment mean (min–max)		Eligibility	
		M	F	M	F	M	F
Regina Margherita Children's Hospital	52	24 (46.2)	28 (53.8)	9 (1–21)	8 (0–19)	21 26	
AOU San Luigi Gonzaga	19	12 (63.15)	7 (36.85)	41 (21–67)	34 (21–53)	12	6
San Giovanni Bosco Hospital	31	17 (54.8)	14 (45.2)	51 (22–77)	52 (19–67)	15	13
AOU Molinette Hospital	7	4 (57.2)	3 (42.8)	27 (18–35)	45 (32–55)	4	3
ASL CN1	15	10 (66.7)	5 (33.3)	58 (31–73)	53 (30–73)	8	3
ASL AL	6	4 (66.7)	2 (33.3)	46 (23–77)	57 (46–67)	3	2
CTO 2	1 (50)	1 (50)	30 (NA)	60 (NA)	1	0	
AO Ordine Mauriziano of Torino	4	1 (25)	3 (75)	37 (NA)	46 (27–57)	0	3
ASL TO3	6	1 (16.7)	5 (83.3)	51 (NA)	76 (72–83)	1	3
ASL TO4	9	7 (77.8)	2 (22.2)	54 (20–69)	48 (45–51)	5	1
SS of genetics Cuneo	3	2 (66.7)	1 (33.3)	56 (54–57)	45 (NA)	2	1
ASL VCO	4	4 (100)	0 (0)	45 (24–57)	NA	4	NA
ASL NO	2	2 (100)	0 (0)	33 (21–45)	NA	2 NA	

List of recruitment centres (Nephrology Units and Genetics Units) in Piedmont Region, and main features of the cohort included in the present study (n = 160). Number of cases, age at recruitment (mean age, min and max age) and eligibility for NGS are listed divided by gender (M: male; F: female). Recruiting centres are: San Giovanni Bosco Hospital; Regina Margherita Children’s Hospital; AOU San Luigi Gonzaga: Azienda Ospedaliera Universitaria San Luigi Gonzaga; AOU Molinette Hospital: Azienda Ospedaliera Universitaria Molinette Hospital; ASL CN1: Azienda Sanitaria Locale—Cuneo, Mondovì and Savigliano; Struttura Semplice Genetics and Molecular Biology, ASL CN1 – Cuneo:ASL AL: Azienda Sanitaria Locale—Alessandria; CTO: Centro Traumatologico Ortopedico; ASL TO3: Azienda Sanitaria Locale—Collegno and Pinerolo; ASL TO4: Azienda Sanitaria Locale—Ciriè, Chivasso and Ivrea; ASL VCO: Azienda Sanitaria Locale del Verbano Cusio Ossola; AO Ordine Mauriziano di Torino: Azienda Ospedaliera Ordine Mauriziano di Torino; ASL NO: Azienda Sanitaria Locale di Novara

All patients included in the study provided written informed consent.

### Set-up of the platform for genetic diagnosis of kidney diseases potentially leading to organ failure

**Fig. 1:**
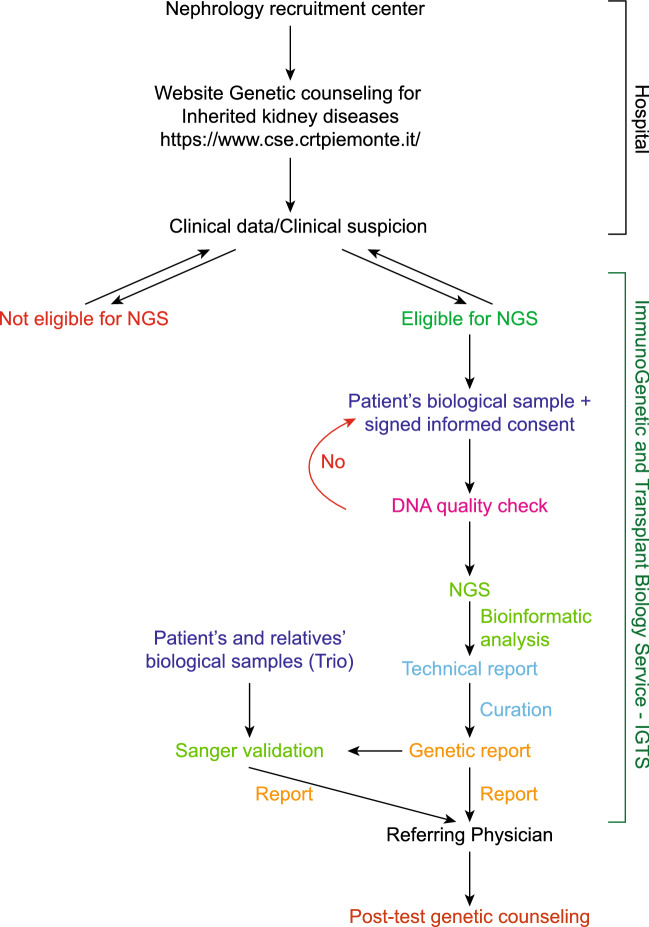
Flowchart of the genetic counselling for inherited kidney diseases. Patients are recruited from the nephrology centres and clinical data are shared with the ImmunoGenetics and Transplant Biology Service (IGTS) through the website for genetic counselling for inherited kidney diseases. Eligibility is assessed based on familiarity, clinical suspicion, and available exams. For eligible patients, a biological sample is processed for NGS analysis. A genetic report is generated and then sent back to the referring physician. The last step provided by the Service is post-test genetic counselling

We set up a web-based genetic service to provide initial genetic counselling to support regional nephrology centres in Piedmont that requested genetic evaluation (Fig. 1). Whenever possible, the referral centre provided IGTS with the patient's medical records including a detailed family history, clinical data from routine diagnostic procedures, parameters of kidney function, imaging data and biopsy results (https://www.cse.crtpiemonte.it/auth/CRT%20LoginGENnew.html).

The platform allowed remote multidisciplinary consultation in order to decide whether patients were eligible for this type of genetic test and to allocate patients with CKD into one of the following categories:

- Patients with a positive family history for CKD;
- Patients for whom genetic confirmation of the clinical diagnosis was required;
- Patients with CKD with no clinical diagnosis of a definite disease.

### Genetic testing

DNA was extracted from blood samples, evaluated for integrity, and then processed for NGS analysis. Sequencing data were analysed by bio-informatics tools to identify, annotate and prioritize variants in order to generate a technical report. Variants were included in the genetic report that was then shared with the referring physician. Sanger sequencing on a second independent DNA extraction was performed to confirm NGS results. When possible, family segregation studies were performed.

The outcome of the genetic test was shared with the clinical team to plan the following steps (Fig. 1). Patients were referred to the closest genetic counselling centre.

### Diagnostic cohort

The validity of the platform and of the analytical pipeline was tested in a training cohort of 29 blindly tested patients for whom clinical and genetic diagnoses were already available. In each case previous genetic diagnosis was confirmed, suggesting that the adopted workflow is effective in the identification of monogenic kidney disease causative variants.

Of the 160 patients for whom genetic analysis was requested, 22 were excluded after a second re-evaluation (due to older age or confounding co-morbidities), while 138 were eligible for NGS analysis. Among them, 52 were children (< 18 years old, 37.7%), while 86 were adults (62.3%). Seventy-eight/138 (56%) were male [24 in the paediatric (46.2%) and 54 (62.8%) in the adult cohort, Table 2]. Sixty-seven out of 138 patients (48.5% in total; 34.6% in the paediatric and 57.0% in the adult subset) had a positive family history for kidney disease (Table 2).

**Table 2:** Characteristics of patients eligible for NGS

Eligible cohort (n = 138)		
Features	Paediatric (n = 52)	Adults (n = 86)
Sex		
Female n (%)	28 (53.8)	32 (37.2)
Male n (%)	24 (46.2)	54 (62.8)
Positive family history n (%)	18 (34.6)	49 (57.0)
Age at onset mean (min–max)	3 (0–14)	37 (0–80)
Clinical suspicion		
CAKUT n (%)	3 (5.8)	0 (0)
Tubular disease n (%)	5 (9.6)	6 (7)
Ciliopathies n (%)	13 (25)	19 (22.1)
Nephrolithiasis/nephrocalcinosis n (%)	1 (1.9)	1 (1.2)
Glomerular disease n (%)	9 (17.3)	12 (13.9)
Haemolytic uraemic syndrome n (%)	1 (1.9)	3 (3.5)
Organ failure for unknown reasons n (%)	18 (34.6)	42 (48.8)
Others n (%)	2 (3.9)	3 (3.5)
Genetic diagnosis		
Cases with variants identified and in line with the clinical phenotype	30 (57.7)	48 (55.8)
Cases with no variants identified or incompatible with the clinical phenotype	22 (42.3)	38 (44.2)
CKD stage		
I	39 (75)	34 (39.6)
II	6 (11.5)	8 (9.3)
III	2 (3.9)	15 (17.5)
IV	1 (1.9)	11 (12.8)
V	0 (0)	10 (11.6)
Dialysis	0 (0)	4 (4.6)
Transplanted	4 (7.7)	4 (4.6)
Kidney biopsy performed	15 (28.8)	29 (33.7)
Imaging	41 (78.8)	69 (80.2)
Glomerular filtration rate (ml/min/1.73 m^2^)		
≥ 90	39 (75)	34 (39.6)
60–89	6 (11.5)	7 (8.1)
30–59	2 (3.9)	16 (18.6)
15–29	1 (1.9)	12 (13.9)
< 15	4 (7.7)	17 (19.8)
Other characteristics		
Diabetes	0 (0)	5 (5.8)
Hypertension	8 (15.4)	60 (69.8)
Extra-renal features	21 (40.4)	28 (32.6)

Clinical details of the NGS-eligible study cohort (138 patients). Eligible patients are sub-divided on the basis of their gender, presence of a positive family history for kidney diseases, age at onset (mean, min and max age), clinical suspicion provided by clinicians at recruitment, results from genetic testing, chronic kidney disease (CKD) stage, availability of kidney biopsy or imaging data, glomerular filtration rate and other features. Number and percentage of cases are shown. CAKUT: congenital abnormalities of kidney and urinary tract

Among the patients who were eligible for NGS, clinical suspicions were as follows: 32 (23.2%) patients who presented with clinical features compatible with ciliopathy, with or without liver involvement, most of whom (30/138; 21.7%) were diagnosed with polycystic kidneys; 21 patients (15.2%) who presented with a suspected glomerular disease, 11 (8%) with tubular diseases, 2 (1.4%) with nephrolithiasis/nephrocalcinosis, and 4 (2.9%) with haemolytic uraemic syndrome (HUS). Notably, a considerable percentage of these patients (60, 43.4%) presented with organ failure of unknown origin (Table 2). As expected, the great majority of paediatric patients (75%) were in stage I CKD, with a glomerular filtration rate ≥ 90 ml/min/1.73 m^2^. Conversely, the majority of adult patients were in stages III-IV and presented important comorbidities, including diabetes (5.8%) and hypertension (69.8%) (Table 2).

### Clinical exome sequencing and raw data processing

Libraries were prepared using the TruSight One Expanded Sequencing Kit (Illumina, San Diego, CA, USA) following the manufacturer's instructions. Raw data were processed as reported in the Supplemental Methods (Online Resource). The choice of the clinical exome approach was dictated by the experimental need of performing a single sequencing, followed by flexible in silico analysis of organ-specific gene panels (e.g. kidney or liver), further tailored on the basis of the clinical suspicion, if available. The list of causative genes associated with kidney disease is updated twice a year, with eventual re-analysis of patients with negative genetic reports every 24 months, without the need for DNA re-sequencing.

### Design of an ad hoc pipeline of analysis to identify causative genes

To perform variant calling and identify causative variants, we designed an ad hoc pipeline of analysis based on sequential inclusion/exclusion steps. After reads were aligned to the GRCh37 as the reference genome using BWA, Isaac Aligner, GATK tools from Illumina, variants were processed using Variant Interpreter software, filtering-in mutations on the basis of a phenotype to genotype correlation. To this end, we generated a Clinical Phenotype to Genotype (CPTG) database by reviewing data from the literature and the main databases on inherited/orphan diseases (e.g., Online Mendelian Inheritance in Man-OMIM, Orphanet, PanelApp). CPTG associates clinical phenotypes to causative genes and is restricted to a panel of 225 genes related to kidney diseases (Supplementary Table 1, Online Resource). Variants were firstly filtered by using gene lists tailored on the basis of clinical suspicion, if available. If a pathogenic or a likely pathogenic variant was identified, analysis was stopped, otherwise, all 225 genes were tested. Synonymous variants were filtered out, while inclusion criteria for the remaining variants (non-synonymous, frame shift, splice site, non-sense) were (1) coverage ≥ 20 ×, (2) frequency ≥ 0.3 and (3) frequency in the overall population ≤ 1% (to exclude polymorphisms which at the time of analysis were not known to be associated with clinical phenotype of kidney diseases), as reported in the 1000 Genomes Project (1 KG) and Exome Aggregation Consortium (ExAC) databases.

Inheritance mode was considered next. Specifically, if heterozygous mutations were found in genes associated with autosomal recessive (AR) diseases, they were carefully re-analysed to check for variants in genes known to be responsible for clinical phenotype in association with other genes (digenic diseases).

Filtered variants were then annotated (1) on the basis of the main public databases reporting associations between gene variants and clinical phenotype, including OMIM, ClinVar, Human Genome Mutation Database (HGMD), the Genome Aggregation Database (GnomAD), database of single nucleotide polymorphism (dbSNP), Varsome, Exome Variant server (EVS) and (2) by considering the impact on protein structure or function by in silico prediction tools.

**Fig. 2:**
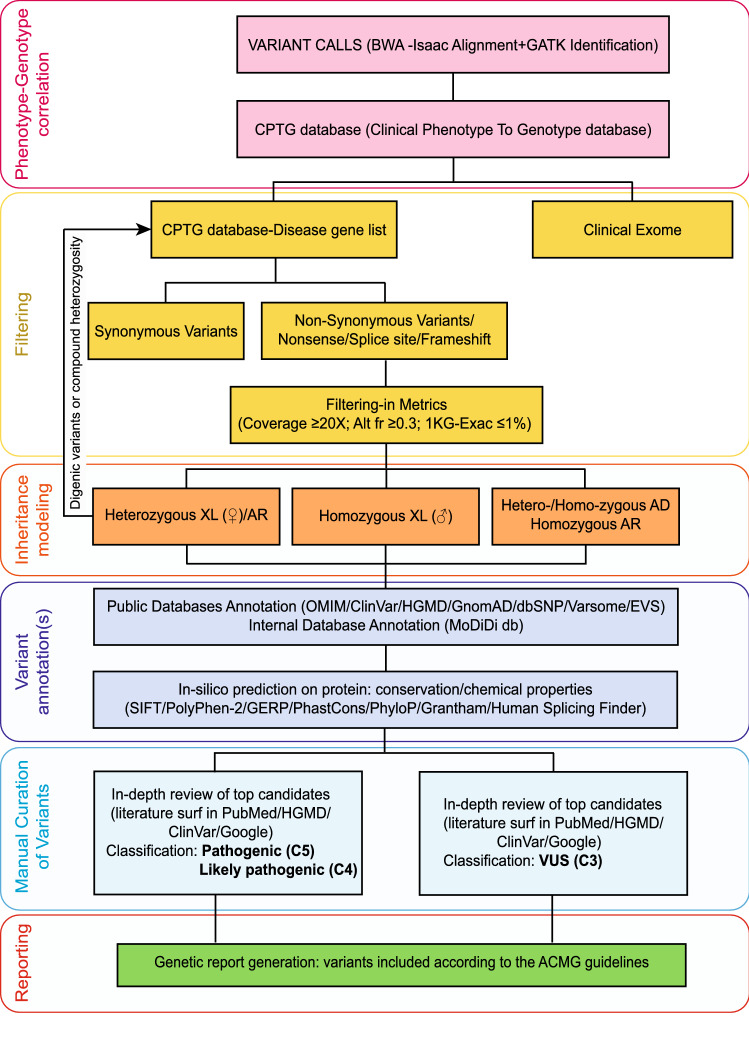
Ad hoc pipeline of analysis. The pipeline is made up of several consecutive steps: phenotype-genotype correlation, filtering-in based on type of variant/frequency and disease list, inheritance model, variant annotation(s), manual curation and reporting of variants. For each step, specific actions and tools are indicated. *BWA* Burrows–Wheeler aligner, *GATK* genome analysis toolkit, *CPTG* clinical phenotype to genotype database, *Alt fr* altered allele frequency, *1 KG* 1000 Genomes Project, *ExAC* Exome Aggregation Consortium, *OMIM*: online mendelian inheritance in men, *HGMD* human genome mutation database, *GnomAD* the genome aggregation database, *dbSNP* database of single nucleotide polymorphism, *EVS* exome variant server

Variants classified as “pathogenic C5” and “likely pathogenic C4” were always included in the genetic report, as were “variants of unknown significance (VUS) C3” in genes associated with diseases with autosomal dominant (AD) or X-linked recessive (in males) mode of inheritance, while C3 variants in genes associated with diseases having AR mode of inheritance were reported only if they were in line with the clinical phenotype (Fig. 2). Confirmation by Sanger sequencing and family segregation studies were performed whenever possible.

Classification of the identified variants and their description in the genetic report were in line with The American College of Medical Genetics and Genomics (ACMG) policy statement on clinical sequencing (https://www.acmg.net/) and with the Italian Society of Human Genetics (SIGU) [16].

### Costs related to the NGS approach for the diagnosis of genetic kidney diseases

Overall, the cost of analysis per sample was differentiated on the basis of clinical suspicion: if a specific disease with < 3 causative genes was suspected the cost charged to the national health system was 1062 euros. For all other cases the cost charged to the national health system was 2262 euros.

## Results

### Overall genetic findings

Overall, by adopting the reported bio-informatics analysis pipeline, we detected 129 variants in 65 genes, with 28 patients carrying more than one variant. Interestingly, of all these variants, only 3 were recurrently present in more than one patient, while all the others were uniquely carried by individual patients.

**Fig. 3:**
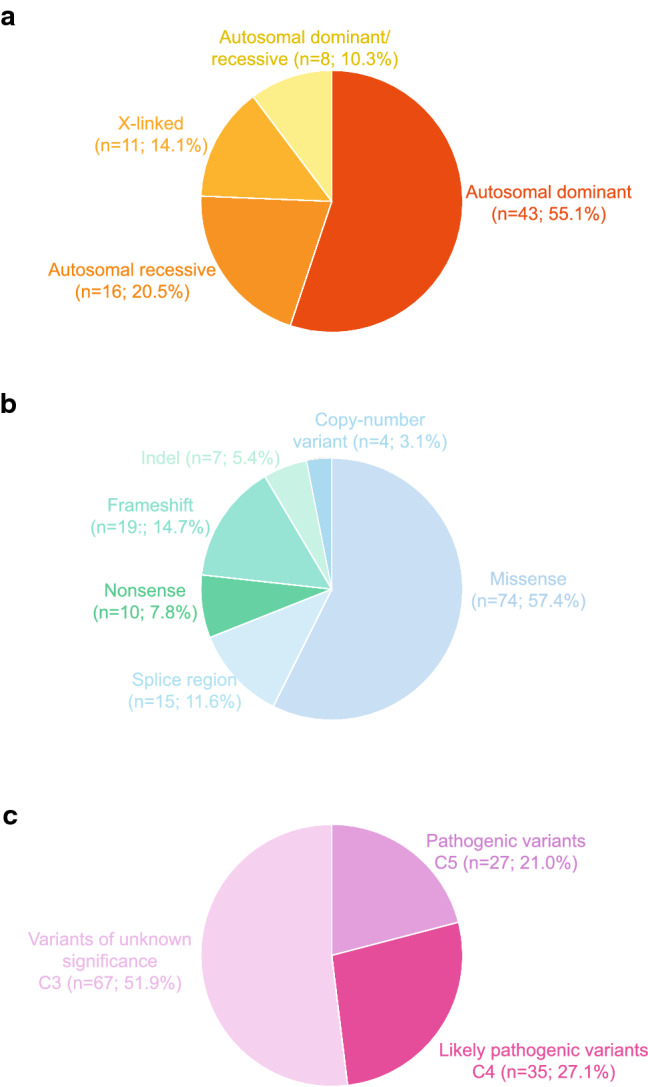
Classification of the identified variants in the Piedmont cohort. **a** Number and percentage of patients having an autosomal dominant, autosomal recessive or X-linked disease on the basis of NGS-identified variants. **b** Classification of the identified variants as missense, nonsense, frameshift, insertion/deletion (indel) or affecting the splice site. Copy number variants (CNVs) are also represented. Number and percentage of variants belonging to the various categories is indicated in brackets. **c** Number and percentage of variants classified on the basis of the American College of Medical Genetics guidelines, considering pathogenic C5, likely pathogenic C4 and variants of unknown significance (VUS, C3)

Genetic variants were classified according to ACMG guidelines. In 78/138 (56.5%) patients, at least one variant was compatible with the clinical phenotype, as indicated in Table 3. In the remaining (60/138; 43.5%) patients, variants were either not present, or heterozygous in autosomal recessive genes or they were not in line with the clinical phenotype (not shown). Among patients for whom we identified variants compatible with the phenotype, 43 (55.1%) presented heterozygous variants in genes associated with autosomal dominant diseases, 16 (20.5%) were homozygous or compound heterozygous with variants in genes associated with autosomal recessive disease (among which 1 was a copy number loss) and 11 (14.1%) were characterized by variants in genes mapping on chromosome X (among which 2 were copy number losses). Lastly, 8 patients (10.3%) presented with variants in genes with both an autosomal dominant and autosomal recessive mode of inheritance (Table 3; Fig. 3a).

**Table 3:**
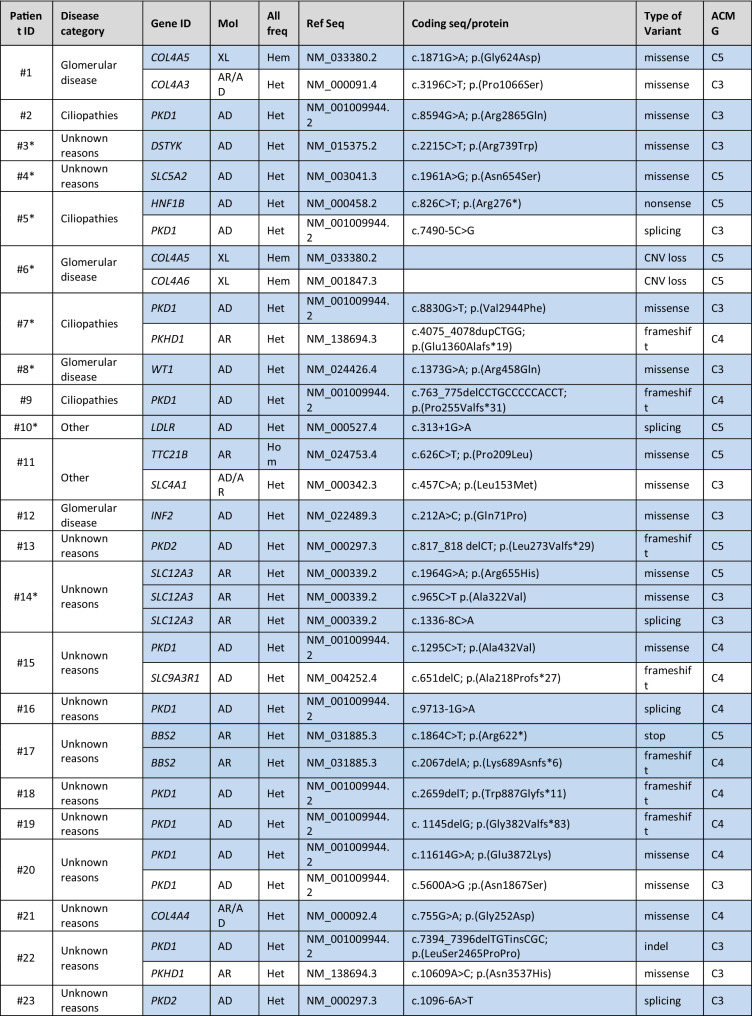

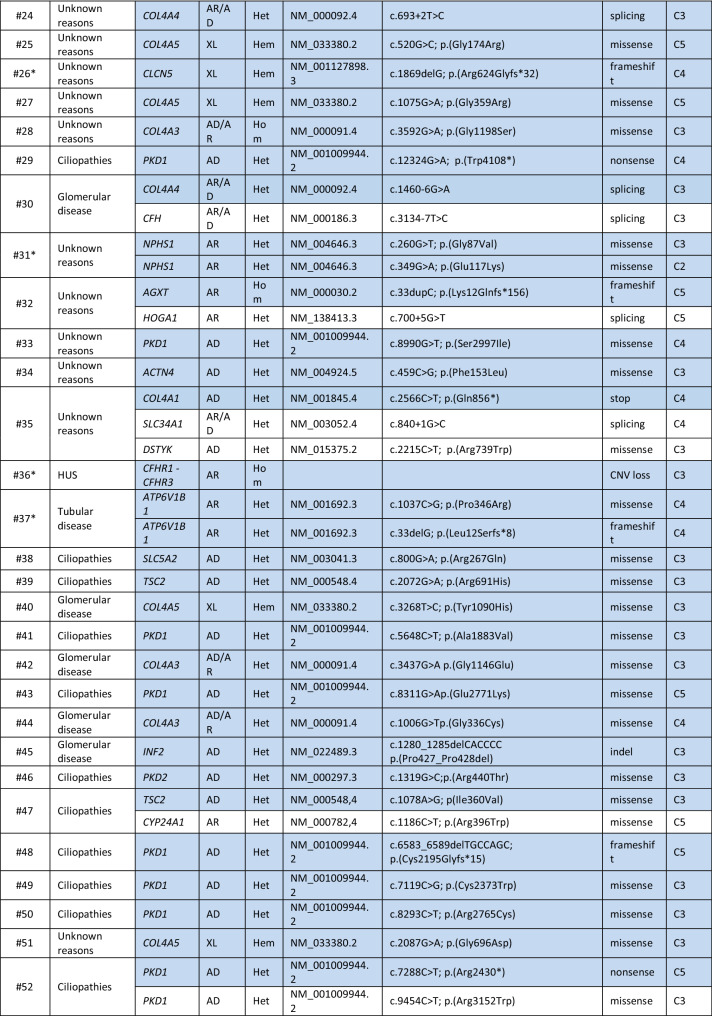

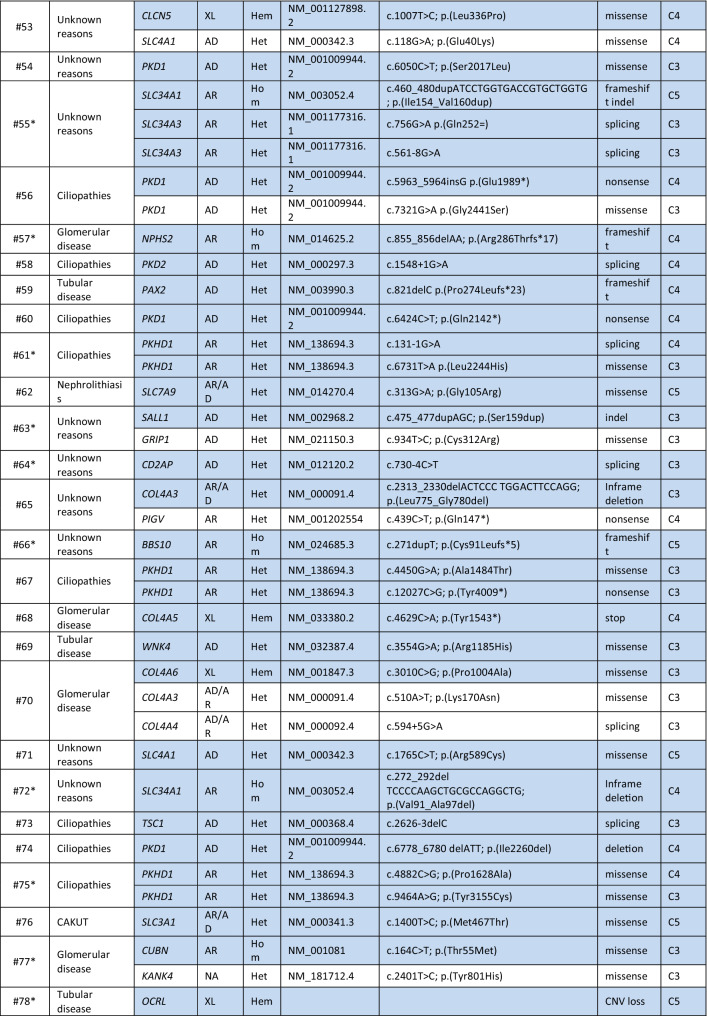
List of potentially diagnostic genetic variants

The table shows the list of 78 patients in whom a potentially diagnostic genetic variant may be present Asterisk**s** indicate the family segregation studies that were carried out. When more than one variant is present, the ones potentially explaining the clinical phenotype are highlighted in blue

*MoI* mode of inheritance, *All. Freq* allele frequency, *Ref Seq* reference sequence, *AD* autosomal dominant, *AR* autosomal recessive, *XL* X-linked, *Het* heterozygous, *Hom* homozygous, *Hem* hemizygous, *CNV* copy number variation, *Indel* insertion/deletion, *HUS* haemolytic uraemic syndrome

When considering all 129 variants in our cohort, 74 (57.4%) were missense, 10 (7.8%) nonsense, 19 (14.7%) frameshift, 7 (5.4%) indel, 15 (11.6%) variants affected the splicing regions and 4 (3.1%) were copy number variants (Fig. 3b).

Furthermore, when classifying all the variants identified by clinical exome sequencing according to ACMG guidelines to describe mutations in genes that cause Mendelian disorders, we found 27 variants defined as “pathogenic C5” (21.0%), 35 as “likely pathogenic C4” (27.1%) and 67 as “variants of unknown significance C3” (51.9%) (Table 3; Fig. 3c), considering that 28 patients were characterized by the presence of more than one variant with different classification.

### Association between clinical and molecular diagnosis

**Fig. 4:**
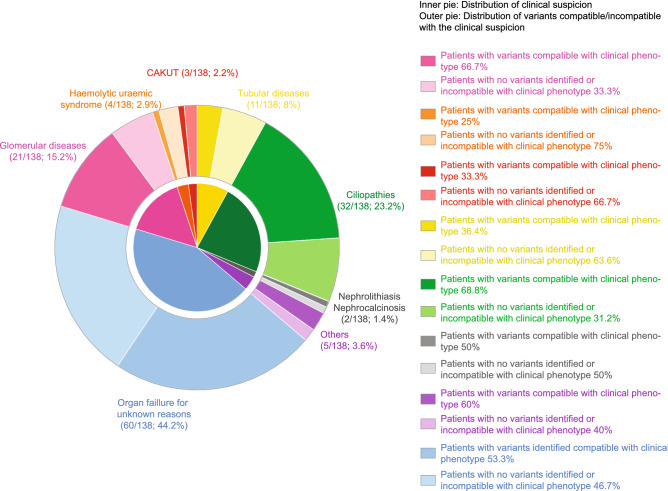
Clinical and genetic diagnosis in the Piedmontese CKD cohort. Patient cohort is divided on the basis of the clinical suspicion (inner pie). Number and percentage of patients for each macro-category are indicated outside the outer pie, which instead represents the percentage of patients with identified causative variants (variants in line with the clinical phenotype) and patients with no causative variants identified or variants incompatible with the clinical phenotype for each disease category. Specific percentages of these cases are reported on the right with a colour-code legend

The diagnosed cases, defining patients for whom genetic variants in line with the clinical phenotype were identified, were differentially distributed when considering the clinical suspicion categories (Table 3; Fig. 4). A high detection rate was obtained in glomerular diseases (14/21 cases; 66.7%), especially Alport disease and ciliopathies (22/32 cases; 68.8%), particularly ADPKD, while for tubular diseases and HUS, causative variants were identified in 4 out of 11, and 1 out of 4 cases, respectively. In the nephrolithiasis and nephrocalcinosis subset, one patient presented with a potentially causative variant in a relevant gene. With regard to the remaining categories, phenotype-related variants were detected in 50% of cases (4 out of 8). Moreover, our NGS approach identified the genetic culprit in a significant proportion of cases presenting with organ-failure of unknown origin (32/60 cases; 53.3%).

Among the cohort of patients with variants identified by NGS, all cases were validated by Sanger sequencing performed on a second independent aliquot of DNA. When possible, specifically in 23/52 paediatric patients, variant(s) were validated in the proband and in the trio. This analysis confirmed the segregation of variants in the family and helped clarify the clinical significance of “C3 VUS”.

## Discussion

In this study we describe an ad hoc-designed web-based platform built to connect regional Nephrology and Genetics centres to a centralized facility that provides genetic testing for patients with CKD. Herein we share our preliminary 15-month experience in applying this targeted sequencing to achieve a genetic diagnosis for undiagnosed patients with CKD in a well-selected cohort of patients from north-west Italy.

Our study presents two points that are worthy of interest. First, the feasibility of a centralized platform to support multidisciplinary consultation in patients with a high clinical suspicion of a monogenic condition. Second, an improvement in the diagnostic rate of patients with CKD and no previous definite diagnosis.

Our approach is based on a web-based platform as an extension of the existing regional transplant network. By using this platform, we attempted to optimize multidisciplinary consultations for patients for whom a monogenic condition was suspected. In order to explain the philosophy and the practical issues of the platform two training courses for the nephrologists of the recruiting centres were organized in 2019. Moreover, in order to reduce waiting time, a rapid (mean waiting time of 3 days), web-based, pre-test genetic assessment was offered, with no need for patients to have in-person genetic counselling.

Patients' samples and informed consent were obtained through the Nephrology or Genetic Counselling Services, thus overcoming the need for the patient and his/her family to travel. The connection of the IGTS to the various nephrology Units throughout the Region was made possible by a capillary network of the Regional Centre for Transplantation. To speed up the connection between “the edge” and “the centre” of the hub, an IT platform was set-up allowing clinicians and geneticists to share clinical data and genetic reports. Recruited patients were initially evaluated by geneticists for their eligibility for NGS based on several criteria including family history and clinical data. Analyses of sequencing data and identification of the causative variants were performed based on an ad hoc pipeline.

Close to 300 patients were recruited between September, 2018 and March, 2020, and a final genetic report was available for 160 of them after a median time for genetic analysis of 6 months. The remaining 140 patients were in different steps of the diagnostic process at the time of this interim analysis.

In this study, we performed clinical exome sequencing followed by an in-silico analysis focused on selective genes in a cohort of 138/160 recruited patients affected by CKD. In 56.5% of cases NGS analysis was able to determine the molecular genetic cause of the disease, revealing 129 variants in 65 genes. These results are on average higher than those reported in the literature, likely due to patient pre-selection on the basis of positive family history and clinical suspicion [9].

With regard to the need to provide genetic confirmation of a previous clinical diagnosis, NGS analysis was able to confirm 68.8% of ciliopathies, a percentage that is in line with previous publications [17]. The detection rate was higher in glomerular diseases (66.7% vs. 14% reported in the literature) and nephrolithiasis (50% vs. 15–30%) [9]. This high percentage is due to selection of patients with a suspicion of Alport disease, at least based on biopsy results. In contrast, the percentage of solved cases presenting with Congenital Anomalies of the Kidneys and of the Urinary Tract (CAKUT) and haemolytic uraemic syndrome was quite low, with a considerable number of cases remaining undiagnosed. A reason for these results could be related to either the genetic heterogeneity of the disease, with many causative genes still to be identified, or to non-genetic causes [8].

In a considerable subset of the recruited cohort, patients were referred to genetic analysis because of a kidney disease of unknown origin. As expected, based on previous experience from other centres, this approach proved to be efficient in revealing causative variants: in a significant number of these cases, we were able to identify genetic variants that were in line with the clinical phenotype, thus helping clinicians in the management of these patients. Surprisingly, in our cohort, the percentage of patients for whom a genetic variant in line with the clinical phenotype was identified was not so different when considering paediatric (57.7%) and adult (55.8%) subgroups. One explanation is that our adult cohort was carefully selected for patients with a strong suspicion of an underlying genetic condition. In line with the selection of the cohort is the limited number of cases that were re-classified. Of note, 18 out of 60 patients lacking a definitive diagnosis were children. NGS application to this subgroup appeared to be a useful tool as it resulted in the detection of variants in an appreciable number of cases (10 out of 18; 55.5%), and provided a genetic explanation for their clinical condition.

Establishing a precise genetic diagnosis, especially for childhood-onset CKD, allows for pre-emptive screening for extra-renal manifestations. In some cases, the kidneys are not the only affected organs and variants in selective genes may cause syndromic diseases. In other cases, the phenotype is the result of hypomorphic mutations leading to variable expressivity and thus resulting in varying clinical manifestations. Moreover, it must be kept in mind that some disease-causing genes may manifest as de novo variants, with a non-inherited history. Finally, because of the high phenotype heterogeneity, several forms of IKDs may become evident only later in life, when patients reach ESKD. Establishing an early and accurate diagnosis will result in better patient management, improving quality of life, and avoiding useless treatments. Furthermore, it allows early screening of at-risk family members.

This technical approach has some known drawbacks. In exome sequencing, variants occurring in the intronic and promoter regions cannot be identified, and not all genomic regions are equally covered. Moreover, regions with high guanine-cytosine content, and high sequence homology with pseudogenes may be missed. Even detection of copy number variations or structural variants can be difficult and need to be further validated by alternative approaches. An additional limitation of this type of sequencing is represented by the detection of pathogenic variants in the *MUC-1* gene, represented by duplicated C or inserted A nucleotides within the coding variable-number tandem repeats (VNTRs), which cannot be identified by exome or genome sequencing, but can only be identified by targeted analysis [18]. Finally, we have to underline that some genes known to be associated with specific CKD phenotypes are not included in this clinical exome panel, and thus variants occurring in these genes cannot be investigated. It is also worth pointing out that the list of genes involved in CKD is progressively expanding [9], therefore, applying the updated list of genes in the re-analysis of previously sequenced patients who received a non-conclusive or negative genetic diagnosis may result in the identification of causative genes. Likewise, variants of unknown significance identified by NGS can be re-classified over time, benefiting from periodic updates. These latter observations also justify the choice of the experimental approach adopted in this study based on clinical exome sequencing instead of limited and fixed targeted sequencing panels.

In conclusion, this study shows that clinical exome sequencing is a non-invasive, highly effective tool for genetic diagnosis if the program is supported by careful candidate selection. It can be useful in identifying patients who would benefit from targeted therapies, such as vasopressin 2 antagonists in the case of ADPKD. Furthermore, it may impact on therapy choices, particularly in the case of FSGS, and in the selection of the ideal family member as a kidney donor. This approach is especially applicable in geographic areas where the interaction between a robust nephrological network and genetic facilities is long-standing. Lastly, it can be cost-effective, especially if it is applied early in the diagnostic flow of the patient as it may (1) provide an early diagnosis and (2) avoid unnecessary treatment, while guiding the nephrologist towards the best management of the patient. For all these reasons, this approach could become, in well-characterized cases, an essential step of the diagnostic path.

## Electronic supplementary material

Below is the link to the electronic supplementary material.

Supplementary file1 (DOCX 77 kb)

## References

[1] Canadas-Garre M, Anderson K, Cappa R, Skelly R, Smyth LJ, McKnight AJ, Maxwell AP (2019). Genetic susceptibility to chronic kidney disease—some more pieces for the heritability puzzle. Front Genet.

[2] Chen TK, Knicely DH, Grams ME (2019). Chronic kidney disease diagnosis and management: a review. JAMA.

[3] Satko SG, Freedman BI (2005). The familial clustering of renal disease and related phenotypes. Med Clin N Am.

[4] Skrunes R, Svarstad E, Reisaeter AV, Vikse BE (2014). Familial clustering of ESRD in the Norwegian population. Clin J Am Soc Nephrol.

[5] Connaughton DM, Bukhari S, Conlon P, Cassidy E, O'Toole M, Mohamad M, Flanagan J, Butler T, O'Leary A, Wong L, O'Regan J, Moran S, O'Kelly P, Logan V, Griffin B, Griffin M, Lavin P, Little MA, Conlon P (2015). The Irish kidney gene project-prevalence of family history in patients with kidney disease in Ireland. Nephron.

[6] Freedman BI, Volkova NV, Satko SG, Krisher J, Jurkovitz C, Soucie JM, McClellan WM (2005). Population-based screening for family history of end-stage renal disease among incident dialysis patients. Am J Nephrol.

[7] Connaughton DM, Hildebrandt F (2019). Personalized medicine in chronic kidney disease by detection of monogenic mutations. Nephrol Dial Transplant.

[8] Groopman EE, Marasa M, Cameron-Christie S, Petrovski S, Aggarwal VS, Milo-Rasouly H, Li Y, Zhang J, Nestor J, Krithivasan P, Lam WY, Mitrotti A, Piva S, Kil BH, Chatterjee D, Reingold R, Bradbury D, DiVecchia M, Snyder H, Mu X, Mehl K, Balderes O, Fasel DA, Weng C, Radhakrishnan J, Canetta P, Appel GB, Bomback AS, Ahn W, Uy NS, Alam S, Cohen DJ, Crew RJ, Dube GK, Rao MK, Kamalakaran S, Copeland B, Ren Z, Bridgers J, Malone CD, Mebane CM, Dagaonkar N, Fellstrom BC, Haefliger C, Mohan S, Sanna-Cherchi S, Kiryluk K, Fleckner J, March R, Platt A, Goldstein DB, Gharavi AG (2019). Diagnostic utility of exome sequencing for kidney disease. N Engl J Med.

[9] Mann N, Braun DA, Amann K, Tan W, Shril S, Connaughton DM, Nakayama M, Schneider R, Kitzler TM, van der Ven AT, Chen J, Ityel H, Vivante A, Majmundar AJ, Daga A, Warejko JK, Lovric S, Ashraf S, Jobst-Schwan T, Widmeier E, Hugo H, Mane SM, Spaneas L, Somers MJG, Ferguson MA, Traum AZ, Stein DR, Baum MA, Daouk GH, Lifton RP, Manzi S, Vakili K, Kim HB, Rodig NM, Hildebrandt F (2019). Whole-exome sequencing enables a precision medicine approach for kidney transplant recipients. J Am Soc Nephrol.

[10] Hildebrandt F (2010). Genetic kidney diseases. Lancet.

[11] Ottlewski I, Munch J, Wagner T, Schonauer R, Bachmann A, Weimann A, Hentschel J, Lindner TH, Seehofer D, Bergmann C, Jamra RA, Halbritter J (2019). Value of renal gene panel diagnostics in adults waiting for kidney transplantation due to undetermined end-stage renal disease. Kidney Int.

[12] Mansilla MA, Sompallae RR, Nishimura CJ, Kwitek AE, Kimble MJ, Freese ME, Campbell CA, Smith RJ, Thomas CP (2019). Targeted broad-based genetic testing by next-generation sequencing informs diagnosis and facilitates management in patients with kidney diseases. Nephrol Dial Transplant.

[13] van Nimwegen KJ, van Soest RA, Veltman JA, Nelen MR, van der Wilt GJ, Vissers LE, Grutters JP (2016). Is the $1000 genome as near as we think? A cost analysis of next-generation sequencing. Clin Chem.

[14] Phillips KA, Deverka PA, Hooker GW, Douglas MP (2018). Genetic test availability and spending: where are we now? Where are we going?. Health Aff (Millwood).

[15] Stokman MF, Renkema KY, Giles RH, Schaefer F, Knoers NV, van Eerde AM (2016). The expanding phenotypic spectra of kidney diseases: insights from genetic studies. Nat Rev Nephrol.

[16] Richards S, Aziz N, Bale S, Bick D, Das S, Gastier-Foster J, Grody WW, Hegde M, Lyon E, Spector E, Voelkerding K, Rehm HL, Committee ALQA (2015). Standards and guidelines for the interpretation of sequence variants: a joint consensus recommendation of the American College of Medical Genetics and Genomics and the Association for Molecular Pathology. Genet Med.

[17] Bullich G, Domingo-Gallego A, Vargas I, Ruiz P, Lorente-Grandoso L, Furlano M, Fraga G, Madrid A, Ariceta G, Borregan M, Pinero-Fernandez JA, Rodriguez-Pena L, Ballesta-Martinez MJ, Llano-Rivas I, Menica MA, Ballarin J, Torrents D, Torra R, Ars E (2018). A kidney-disease gene panel allows a comprehensive genetic diagnosis of cystic and glomerular inherited kidney diseases. Kidney Int.

[18] Bleyer AJ, Kmoch S, Adam MP, Ardinger HH, Pagon RA (1993). Autosomal dominant tubulointerstitial kidney disease, *MUC1*-related. GeneReviews®.

