# Supplementary material for: Clinical exome sequencing is a powerful tool in the diagnostic flow of monogenic kidney diseases: an Italian experience
Source: J Nephrol. 2020 Nov 23;34(5):1767–81. doi: 10.1007/s40620-020-00898-8 (PMC8494711; doi:10.1007/s40620-020-00898-8)
Supplement: Supplementary file 1 — Supplementary file1 (DOCX 77 kb) [file 40620_2020_898_MOESM1_ESM.docx]

**Supplemental Methods**

**IT infrastructure**

The web-based platform behind this genetic service was built using the PHP with MYSQL database programming language. The hosting cloud platform is provided by CSI-Piemonte (https://www.csipiemonte.it/web/en), a technological partner of over 100 regional and national public institutions, including healthcare companies and hospitals. To guarantee the security of the stored data, the platform is based on a channel encripted by SSL/TLS. Users’ account is created by the webmaster following a formal request form signed by the medical center director. The web application users are divided in several groups, with different degrees of access, based on the recruitment center and their role. The groups are: 1) medical doctors / peripheral geneticists who send the NGS analysis request; 2) geneticists at Immunogenetic and Biology Transplant Service who evaluate the requests and decide for NGS eligibility; 3) the Immunogenetic and Biology Transplant Service laboratory technicians who accept peripheral blood samples from eligible patients, insert the sample arrival date and then extract DNA; 4) super user, i.e. the webmaster. To guarantee the privacy of recruited patients, group 1 users can only visualize clinical and genetic data of patients recruited in their centers.

**Nucleic acid extraction**

Peripheral blood samples were collected in EDTA tubes and stored at 4 °C. A second blood sample was stored at 4 °C and conserved for Sanger validation. Genomic DNA (gDNA) was extracted from 200 microlitres of blood using DNeasy Blood & Tissue Kit (Qiagen, Hilden, Germany) following the manufacturer’s instructions. Quality and quantity of extracted DNA was assessed by electrophoresis (E-Gel Size Select 2%, Invitrogen, Carlsbad, California, USA), spectrophotometry (NanoDrop 8000, ThermoFisher, Waltham, MA, USA) and fluorimetric assay (Qubit 4 fluorometer, Thermofisher).

Peripheral blood tubes of NGS eligible patients were delivered from recruitment centers to the Immunogenetic and Biology Transplant Service through the regional hospital center couriers, which operate between healthcare centers.

**Clinical exome sequencing and raw data processing**

Libraries were prepared starting from 50 ng of gDNA using TruSight One Expanded Sequencing Kit (Illumina, San Diego, CA, USA) following the manufacturer's instructions. The kit is based on a probe-exome capture approach: gDNA is tagmented, enriched and hybridized to complementary sequences exploiting the Nextera Rapid Capture Enrichment technology by Illumina. The clinical exome sequencing is based on 6,700 genes with clinical relevance, determined on the basis of various public databases (<http://emea.support.illumina.com/content/dam/illumina-marketing/documents/products/datasheets/datasheet_trusight_one_panel.pdf>). Quality check of the enriched libraries was performed on the 2100 Bioanalyzer instrument (Agilent Technology, Santa Clara, CA, USA) using a high sensitivity DNA kit (Agilent Technology, Santa Clara, CA, USA).

Libraries were denatured and diluted as indicated in Denature and Dilute Libraries Guide (NextSeq System, Illumina, San Diego, CA, USA) and sequenced on NextSeq550 benchtop sequencer (High Output v2.5 300 cycles cartridges) using paired-end, 150bp reads flowcells. PhiX control was used as internal control.

Raw data obtained from sequencing were converted to FASTQ files and then aligned with Enrichment 3.1.0 tool (Illumina) and mapped on TruSightOne Expanded v2.0 manifest using Homo Sapiens UCSC GRCh37 genome as reference. The following software programs, BWA-Isaac software, Starling, Canvas and Manta (all from Illumina, San Diego, CA, USA), were used to obtain reads alignment, small structural variants, copy number variants and structural variants calling, respectively. For copy number identification a wild-type DNA was used (NA12878 obtained from the NIGMS Human Genetic Cell Repository at the Coriell Institute for Medical Research, Camden, NJ, USA). Variant annotation was performed by Illumina Annotation Engine and alignment calculation and variant calling metrics obtained by Pluggable Universal Metrics Analyzer (PUMA). This data processing resulted in the generation of BAM files containing aligned reads, variant call files (VCF), SV-VCF files for structural variants and CNVs, PDF report containing enrichment metrics, all used for successive bio-informatics analyses. Reads alignment and exon coverage of genes of interest were checked and displayed by Integrative Genome Viewer, freely available from the Broad Institute, University of California, USA (<https://software.broadinstitute.org/software/igv/>).

**Sanger validation**

The identified variants were analysed by Sanger sequencing to confirm NGS analysis. Briefly, DNA was extracted as reported above starting from a second independent aliquot of proband and/or relative(s)' peripheral blood. Family segregation studies were performed whenever possible to understand whether identified variants were *de novo* variants or not. The DNA regions of interest were amplified by PCR using specific experimental conditions. The purity and specificity of the amplified regions were checked by 1% agarose gel. Amplified PCR products were then Sanger sequenced using the same primers. Electropherograms were then analysed using the Chromas software version 2.6, freely available at [www.technelysium.com.au](http://www.technelysium.com.au/).

**Web Resources**

Online Mendelian Inheritance in Man - OMIM^®^: an online catalogue of human genes and genetic disorders. McKusick-Nathans Institute of Genetic Medicine, Johns Hopkins University (Baltimore, MD); Website URL: <https://omim.org/>

Orphanet**:** an online database of rare diseases and orphan drugs. Copyright, INSERM 1997. Website URL: [http://www.orpha.net](http://www.orpha.net/).

Genomics England PanelApp: a publicly-available knowledgebase that allows virtual gene panels related to human disorders to be created, stored and queried. Website URL: [https://panelapp.genomicsengland.co.uk](https://panelapp.genomicsengland.co.uk/). Specific panels used to update the gene list matching causative genes to kidney diseases were: CAKUT (Version 1.39), Cystic kidney disease (Version 2.0), Tubulo-interstitial kidney disease (Version 1.0), Unexplained kidney failure in young people (Version 1.73).

Exome Variant Server: a database of exome sequencing data. NHLBI GO Exome Sequencing Project (ESP), Seattle, WA. Website URL: <http://evs.gs.washington.edu/EVS/>.

ClinVar: freely available archive of reports of relationships among medically important variants and phenotypes. Website URL: <https://www.ncbi.nlm.nih.gov/clinvar/>.

Human Genome Mutation Database (HGMD): a database of all published gene lesions responsible for human inherited disease[1].

Genome Aggregation Database (GnomAD): a database of exome and genome sequencing data from a wide variety of large-scale sequencing projects. GnomAD flagship paper: <https://www.biorxiv.org/content/10.1101/531210v3>.

Single Nucleotide Polymorphism database (dbSNP): a database of human single nucleotide variations, microsatellites, and small-scale insertions and deletions along with publication, population frequency, molecular consequence, and genomic and RefSeq mapping information for both common variations and clinical mutations [2].

VarSome: variant knowledge community, data aggregator and variant data discovery tool [3].

MutationTaster: free web-based application to evaluate DNA sequence variants for their disease-causing potential [4].

Sorting Intolerant From Tolerant (SIFT): free online application based on sequence homology and the physical properties of amino acids[5].

PolyPhen-2: tool which predicts possible impact of an amino acid substitution on the structure and function of a human protein using straightforward physical and comparative considerations.

Human Splicing Finder (Version 3.1): free online tool to calculate the consensus values of potential splice sites and search for branch points[6].

**Bibliography**

1. Stenson PD, Mort M, Ball EV, Evans K, Hayden M, Heywood S, Hussain M, Phillips AD, Cooper DN (2017) The Human Gene Mutation Database: towards a comprehensive repository of inherited mutation data for medical research, genetic diagnosis and next-generation sequencing studies. Hum Genet 136 (6):665-677. doi:10.1007/s00439-017-1779-6

2. Sherry ST, Ward MH, Kholodov M, Baker J, Phan L, Smigielski EM, Sirotkin K (2001) dbSNP: the NCBI database of genetic variation. Nucleic Acids Res 29 (1):308-311. doi:10.1093/nar/29.1.308

3. Kopanos C, Tsiolkas V, Kouris A, Chapple CE, Albarca Aguilera M, Meyer R, Massouras A (2019) VarSome: the human genomic variant search engine. Bioinformatics 35 (11):1978-1980. doi:10.1093/bioinformatics/bty897

4. Schwarz JM, Cooper DN, Schuelke M, Seelow D (2014) MutationTaster2: mutation prediction for the deep-sequencing age. Nat Methods 11 (4):361-362. doi:10.1038/nmeth.2890

5. Vaser R, Adusumalli S, Leng SN, Sikic M, Ng PC (2016) SIFT missense predictions for genomes. Nat Protoc 11 (1):1-9. doi:10.1038/nprot.2015.123

6. Desmet FO, Hamroun D, Lalande M, Collod-Beroud G, Claustres M, Beroud C (2009) Human Splicing Finder: an online bioinformatics tool to predict splicing signals. Nucleic Acids Res 37 (9):e67. doi:10.1093/nar/gkp215

7. Hildebrandt F (2010) Genetic kidney diseases. Lancet 375 (9722):1287-1295. doi:10.1016/S0140-6736(10)60236-X

**Supplementary Table 1 Clinical Phenotype to Genotype (CPTG) database.** List of kidney pathological phenotypes and the 225 relative causative genes, used for NGS analysis. All kidney diseases are reported and divided in macro-categories, according to the current literature [7]. Mode of inheritance is reported for each disease. AD: autosomal dominant inheritance; AR: autosomal recessive inheritance; XL: X-linked inheritance.
